# Patients’ Willingness to Provide Their Clinical Data for Research Purposes and Acceptance of Different Consent Models: Findings From a Representative Survey of Patients With Cancer

**DOI:** 10.2196/37665

**Published:** 2022-08-25

**Authors:** Anja Köngeter, Christoph Schickhardt, Martin Jungkunz, Susanne Bergbold, Katja Mehlis, Eva C Winkler

**Affiliations:** 1 Section for Translational Medical Ethics Department of Medical Oncology National Center for Tumor Diseases, Heidelberg University Hospital Heidelberg Germany; 2 Section for Translational Medical Ethics National Center for Tumor Diseases German Cancer Research Center Heidelberg Germany; 3 Epidemiological Cancer Registry Baden-Württemberg German Cancer Research Center Heidelberg Germany

**Keywords:** secondary use, consent, data sharing, data access, research benefit and control of data, health data, clinical data, private sector, international data sharing, patient perspective

## Abstract

**Background:**

Secondary use of clinical data for biomedical research purposes holds great potential for various types of noninterventional, data-driven studies. Patients’ willingness to support research with their clinical data is a crucial prerequisite for research progress.

**Objective:**

The aim of the study was to learn about patients’ attitudes and expectations regarding secondary use of their clinical data. In a next step, our results can inform the development of an appropriate governance framework for secondary use of clinical data for research purposes.

**Methods:**

A questionnaire was developed to assess the willingness of patients with cancer to provide their clinical data for biomedical research purposes, considering different conditions of data sharing and consent models. The Cancer Registry of the German federal state of Baden-Württemberg recruited a proportionally stratified random sample of patients with cancer and survivors of cancer based on a full census.

**Results:**

In total, 838 participants completed the survey. Approximately all participants (810/838, 96.7%) showed general willingness to make clinical data available for biomedical research purposes; however, they expected certain requirements to be met, such as comparable data protection standards for data use abroad and the possibility to renew consent at regular time intervals. Most participants (620/838, 73.9%) supported data use also by researchers in commercial companies. More than half of the participants (503/838, 60%) were willing to give up control over clinical data in favor of research benefits. Most participants expressed acceptance of the broad consent model (494/838, 58.9%), followed by data use by default (with the option to opt out at any time; 419/838, 50%); specific consent for every study showed the lowest acceptance rate (327/838, 39%). Patients expected physicians to share their data (763/838, 91.1%) and their fellow patients to support secondary use with their clinical data (679/838, 81%).

**Conclusions:**

Although patients’ general willingness to make their clinical data available for biomedical research purposes is very high, the willingness of a substantial proportion of patients depends on additional requirements. Taking these perspectives into account is essential for designing trustworthy governance of clinical data reuse and sharing. The willingness to accept the loss of control over clinical data to enhance the benefits of research should be given special consideration.

## Introduction

### Background

Secondary use of clinical data for biomedical research purposes has great potential for various types of noninterventional, data-driven studies. We define secondary use of clinical data as the collection and reuse of clinical data in data gathering, noninterventional biomedical research, or learning activities; clinical data are collected during and for the purpose of patient care [1]. Research using clinical data has the ethical and efficiency advantages of not requiring additional physical interventions or collection of additional data. Although secondary use aims at improving biomedical knowledge and, in turn, medical care, it does not imply a direct benefit for the patient who has released their data.

The blurring of the boundaries between research and care, as envisaged in concepts of learning health care systems, is currently visible only in few areas [1,2]. The endeavor to merge these different system logics is faced with emerging challenges such as limited utility of specific consent models for research or false expectations regarding their benefits on the part of patients [3]. The goal of this paper was to contribute the patients’ perspective to the debate and potential solutions to the current challenges of secondary use of clinical data in the context of learning health care systems.

Previous studies with citizens and patients have already shown that certain aspects seem to be crucial for supporting secondary use, such as who conducts the research (eg, academic or commercial), whether data are transferred to other countries, and what consent model is applied [2-9]. However, owing to varying research designs, for example, by examining different study units, applying different survey instruments, and being conducted in diverse health care systems, these studies, taken together, have heterogeneous results.

Consent is a crucial component of respecting patient autonomy and building trust in health research. However, the specific consent paradigm of clinical trials cannot easily be applied to the secondary use of clinical data because most scientific questions are unknown at the time consent is obtained, that is, when the patient receives care. Newly applied models for secondary use of clinical data, such as broad consent or data use by default (with the option to opt out any time), facilitate research with clinical data, but are criticized from an informational self-determination perspective for offering patients insufficient control over their clinical data. However, previous studies have identified patients’ and citizens’ openness toward these new models [5,10-12]. Other empirical studies show that, to increase research benefits, participants seem willing to accept the loss of control over their data [13-16]. However, no studies have yet been conducted to assess the acceptance of consent models in light of the trade-off between the control of clinical data and research utility.

### Aim

The objective of this study was to assess (1) patients’ general willingness and relevant requirements to share pseudonymized clinical data for research purposes, (2) acceptance of different consent models including characteristics of data control and research utility, (3) preferences regarding the setting to provide consent, and (4) general expectations toward data use and other stakeholders.

## Methods

### Survey Development

The questionnaire (Multimedia Appendix 1) was based on a review of the relevant scientific literature and a preparatory expert interview study among stakeholder groups engaged or affected by the planned secondary use of clinical data in Germany [17]. In total, 2 representatives for patient interests were included in the expert sample. The questionnaire was developed through several discussion and feedback rounds by the international and interdisciplinary project team, consisting of social scientists; ethicists; legal scholars; and clinicians with expertise in social, ethical, legal, or practical aspects of secondary use of clinical data. To ensure comprehensibility and technical functionality of the questionnaire, cognitive interviews (n=5) with patients with cancer and survivors of cancer who had provided consent were conducted in the pretest phase, resulting in minor adaptions.

To allow participants to develop an informed opinion, the survey included background information about risks and benefits associated with the secondary use of clinical data. The survey consisted of 33 items on the following topics: sociodemographic and disease-related information, expectations and risk perception toward secondary use, willingness to provide clinical data under certain requirements, and acceptability of consent models and procedures. Attitudinal questions were designed as 5-point Likert scale. The survey was approved by the data protection officer of the Heidelberg University Hospital.

### Operationalization of Consent Scenarios

**Figure 1:**
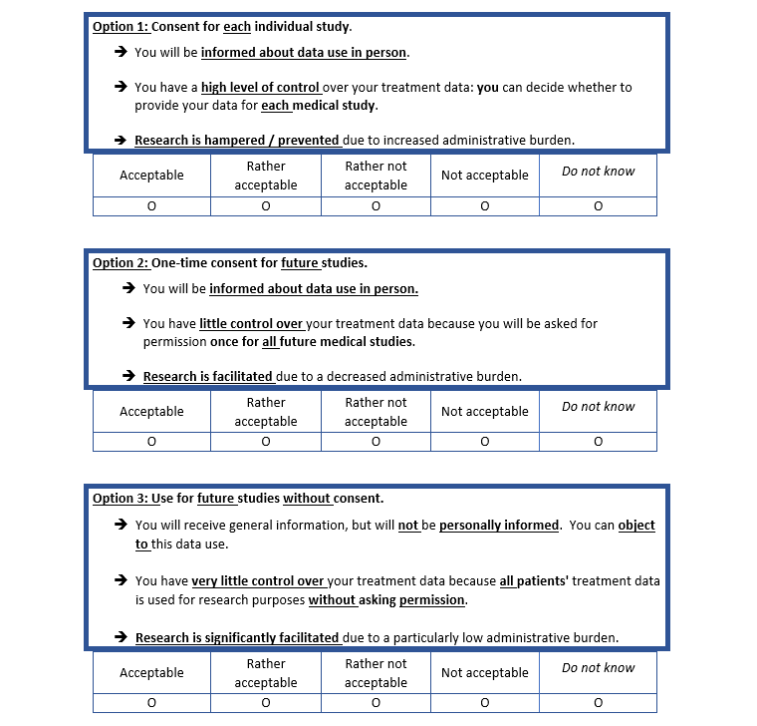
Display of the 3 consent scenarios in the questionnaire (English translation).

In total, 3 vignettes were developed to measure the acceptability of 3 consent scenarios: *specific consent, broad consent*, and *data use by default* (with the option to opt out at any time). Acceptance was measured using a 4-point Likert scale (Figure 1).

Previous studies have reported that participants made a trade-off between research utility and data control [13]. Hence, 3 consent scenarios were designed with information about research utility and control over data. In the process of operationalization, we further reduced the complexity of the theoretical concept to ensure good comprehensibility of the survey material:

1. By *specific consent,* we understand that consent is provided for each individual study (option 1 in Figure 1), as currently performed in clinical trials. Consistent with our preliminary studies [1,17], we inform about high degree of control over the secondary use of clinical data and low research benefit owing to the administrative burden on researchers.
2. In the case of the *broad consent* scenario, 1-time consent is provided for future medical studies with clinical data; moderate control and research utility are presumed (option 2 in Figure 1).
  This vignette refers to the implementation of a broad consent process for the German Medical Informatics Initiative; the development of a unified template for broad consent was accompanied by the German Working Group of Research Ethics Committees [18]. In practice, this model involves safeguards such as the review of each individual research project by a research ethics committee and data access committees, organizational measures to protect patient data, and comprehensive information for patients [18]. To ensure comprehensibility, the details of these safeguards are not provided to the participants of this study.
3. *Data use by default* is use of data for secondary research by default (comparable with Denmark or Estonia) without individual informed consent process, but with the possibility to opt out at any time. This scenario is associated with low degree of data control for patients and facilitation of research as no individual consent needs to be obtained (option 3 in Figure 1).
  Regarding law, the European Union (EU) data protection regulation provides some scope for this scenario of data use based on a legal basis other than informed consent if the potential research benefit clearly outweighs the right to informational self-determination (Art. 9, Paragraph 2, lit. j [19]). Compensating efforts such as ambitious security and privacy measures and extensive general public education about data use and data governance are likely to be ethically and legally necessary. To ensure comprehensibility, the details of these safeguards are not provided to the participants of this study.

### Sampling and Recruitment

The Cancer Registry of the German federal state of Baden-Württemberg sent postal invitations to a random sample of patients with cancer and survivors of cancer, proportionally stratified by age and gender, requesting study participation (n=4219). The sample frame consisted of all registered patients in Baden-Württemberg, Germany, with a diagnosed tumor disease who were aged ≥18 years. Participants had the option of either completing an anonymous and self-administered web-based survey (the hyperlink was provided in the cover letter) or returning an envelope by mail, consenting that their address may be forwarded to the research group to subsequently receive a paper-and-pencil questionnaire. Survey instruments were adapted to the requirements of a mixed-mode survey [20].

Individuals who completed the survey were not compensated.

Data collection occurred from May 2021 to July 2021.

### Analysis

Descriptive statistics were used to express the categorical variables as counts and percentages. Differences in proportions were assessed for statistical significance (*P*<.05) using chi-square tests. The 2-tailed Pearson correlation coefficients were computed. All analyses were performed using SPSS (version 28; IBM Corp).

### Ethics Approval

The study obtained ethics approval from the University of Heidelberg’s research ethics committee (reference number S-361/2018). Informed consent was obtained from the individuals who participated in the study pretest measurement and the written survey.

## Results

### Demographics of Participants

Of the 4155 patients with cancer approached by the Cancer Registry Baden-Württemberg, 838 (20.17%) participants completed the survey. Approximately half of the participants who answered the respective question were women (389/820, 47.4%; Table 1). Of 832 participants, 390 (46.9%) participants were aged between 60 and 74 years, and of 826 participants, 541 (65.5%) participants were retired. In total, 29.8% (247/830) of the participants had a university degree. The most common types of cancer were breast cancer (204/826, 24.7%), prostate cancer (187/826, 22.6%), and gastrointestinal cancer (79/826, 9.6%). The distribution of age, gender, and cancer entity mirrored that of the general distribution of patients with cancer in the Cancer Registry Baden-Württemberg, with minor deviation.

**Table 1:** Demographics of participants.

Characteristics		Values, n (%)
**Gender (n=820)**		
	Women	389 (47.4)
	Men	431 (52.6)
**Age groups (years; n=832)**		
	18-59	186 (22.4)
	60-74	390 (46.9)
	≥75	256 (30.8)
**Highest educational degree (n=830)**		
	Elementary school diploma	84 (10.1)
	Secondary school diploma	398 (47.9)
	Qualification for university entrance	97 (11.7)
	University degree	247 (29.8)
	No school diploma	4 (0.5)
**Employment status (n=826)**		
	Employed or self-employed	219 (26.5)
	Not employed owing to health reasons	45 (5.4)
	Retired	541 (65.5)
	Not employed owing to other reasons	21 (2.5)
**Type of cancer (n=826)**		
	Breast	204 (24.7)
	Prostate	187 (22.6)
	Gastrointestinal	79 (9.6)
	Skin cancer	63 (7.6)
	Non-Hodgkin lymphoma	39 (4.7)
	Lung	31 (3.8)
	Leukemia	22 (2.7)
	Kidney	22 (2.7)
	Head and neck	22 (2.7)
	Uterine or endometrial	21 (2.5)
	Urinary bladder	18 (2.2)
	Stomach	16 (1.9)
	Pancreas	9 (1.1)
	Other	93 (11.3)

### General Willingness to Provide Clinical Data for Biomedical Research Purposes and Requirements for Data Provision

Most participants indicated that they are generally willing to make their clinical data available either without restrictions (527/838, 62.9%) or under certain conditions (283/838, 33.8%). Only 0.7% (6/838) of the participants generally refused to provide clinical data.

Then, the participants who indicated general willingness were asked about certain requirements under which they would provide their clinical data. When asked about the general requirements they deemed relevant, most participants stated the highest possible data security standards (482/838, 57.5%), followed by use of their data for as many research projects as possible (254/838, 30.3%), and being informed about the most important research results (208/838, 24.8%; Multimedia Appendix 2).

Most participants (591/832, 70.5%) stated that they would support research with their data in countries with high level of data protection comparable with German standards; 17.9% (149/832) of the participants stated that they would restrict data use to domestic research projects; and 8.8% (73/832) of the participants agreed to support international projects, independent of the level of data protection (Multimedia Appendix 3).

When asked how long their initial consent should be valid, 38.5% (320/832) of the participants set no time limit and approximately half of the participants demanded to renew consent either after 3 years (181/832, 21.8%), 10 years (227/832, 27.3%), or 30 years (10/832, 1.2%), respectively. In total, 10.2% (85/832) of the participants favored renewal of consent each time their data are used for specific research projects (Multimedia Appendix 4).

A large proportion of participants (532/832, 63.4%) said that they would grant access to researchers, independent of their affiliation; however, 22.7% (189/832) of them did not want to share their data with researchers at for-profit companies that conduct medical research (Multimedia Appendix 5). Only a small proportion opposed the secondary use of their clinical data by their physicians (56/832, 6.7%) or researchers at universities and university hospitals (48/832, 5.8%).

### Acceptance of Consent Models

The questionnaire provided information about 3 consent models that correspond to specific consent, broad consent, and data use by default (with the option to opt out at any time), including the trade-offs of each model between control over clinical data and the facilitation of medical research (Table 2). For each consent model, the participants rated the level of acceptance on a 4-point Likert scale. Each of the 3 consent models showed a medium degree of acceptance with significant mean differences. Of the 838 participants, 491 (58.6%) accepted the broad consent model, 421 (50.2%) accepted data use by default (with the option to opt out at any time), and 323 (38.5%) accepted the specific consent model. Of the 323 participants accepting the specific consent model, 102 (31.6%) did not accept any other model (102/838, 12.2% of the total sample). Sociodemographic characteristics were not significant, except for older participants being more likely to accept data use by default (Pearson coefficient, 2-tailed: *r*=0.138; *P*<.001).

**Table 2:** Acceptance rates of 3 consent models: broad consent, data use by default, and specific consent (N=838)^a^.

Model	Description	Accepted, n (%)	Not accepted, n (%)	Do not know or not answered, n (%)
Broad consent	One-time consent for future studies, informed in person, low level of control, and research is facilitated	491 (58.6)	230 (27.4)	117 (13.9)
Data use by default	Use for future studies without consent process, not personally informed, very low level of control, and research is significantly facilitated	421 (50.2)	347 (41.4)	70 (8.4)
Specific consent	Consent for each study, informed in person, high level of control, and research is hampered	323 (38.5)	372 (44.4)	143 (17.1)

^a^Acceptance was measured using a 4-point scale; results were collapsed into 2 groups (*not acceptable*: not acceptable and rather not acceptable; *acceptable*: acceptable and rather acceptable).

### Preferences Regarding the Setting for Providing Consent

Participants were asked about the most appropriate setting for providing consent for the secondary use of their clinical data for research purposes. Most of them preferred to decide at their general practitioner’s practice (528/838, 63%), and a small proportion of participants preferred to decide during the admission to a hospital (174/838, 20.8%; Multimedia Appendix 6).

When asked about preferred information formats, most participants selected a brief written summary of key points in easy-to-understand language to learn more about secondary use (616/838, 73.5%), followed by face-to-face consultation with physicians (347/838, 41.4%; Multimedia Appendix 7). Participants were asked about who should decide about data access and use by individual research projects: most participants (393/838, 46.9%) favored committees with experts in which the opinion of patients is represented, for example, by patient representatives, whereas a small proportion of participants preferred to leave the decision to an expert committee (without patient representation; 185/838, 22.1%) or to decide for themselves (200/838, 23.9%; Multimedia Appendix 8).

### Concerns in the Event of Data Use

**Figure 2:**
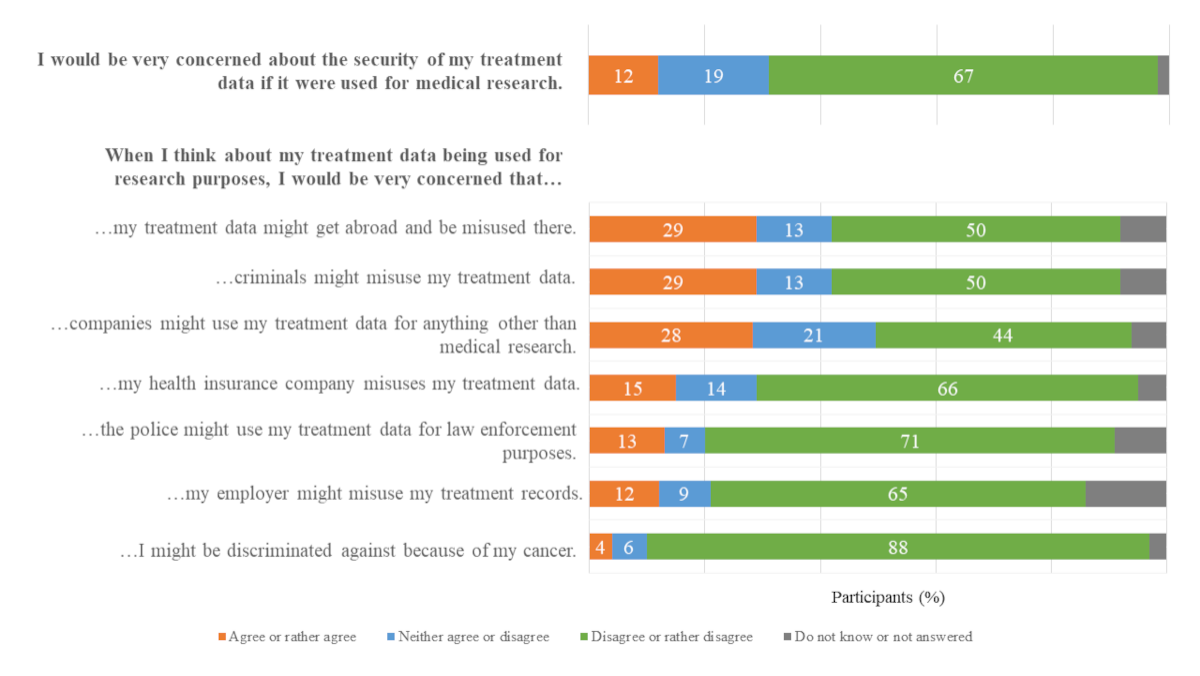
Concerns in the event of data use (N=838).

A small proportion of the participants (99/838, 11.8%) showed major general concerns regarding their clinical data being used for research purposes (Figure 2). Then, all participants were asked about more specific concerns: the largest proportion of participants were worried about the data being misused in countries other than Germany (246/838, 29.4%), data being misused by criminals (244/838, 29.1%), and data being used by companies for something other than medical research (235/838, 28%). Concerns about participants being discriminated against because of cancer were very low (32/838, 3.8%).

### Expectations Toward Benefits, Other Patients, and Physicians

**Figure 3:**
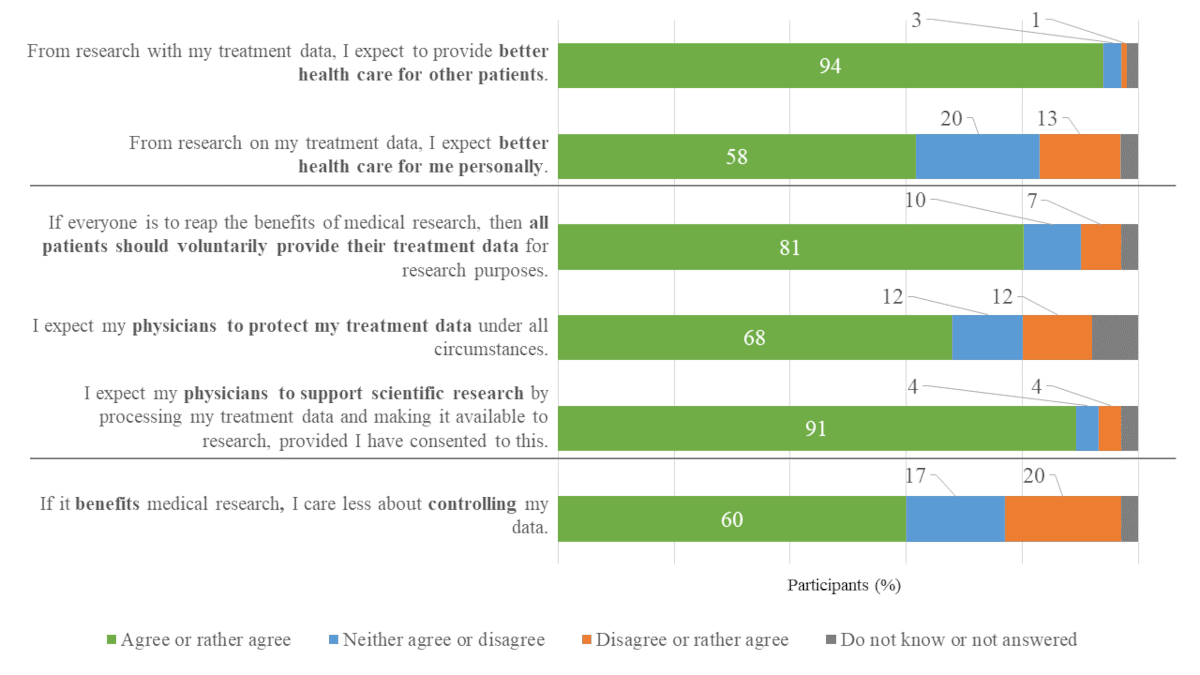
Expectations toward physicians and other patients (N=838).

Approximately all participants (788/838, 94%) expected a benefit for other patients from making their clinical data available for research purposes (Figure 3). More than half of the participants (482/838, 57.5%) mistakenly expected a personal benefit, even though the explanatory text explicitly stated the opposite. Of the 838 participants, 676 (80.7%) participants supported the claim that all patients should voluntarily make their clinical data available for research purposes. In total, 68.3% (572/838) of the participants expected their physicians to protect the participants’ clinical data in all circumstances, and approximately all participants (758/838, 90.5%) expected their physicians to support research, if consent was provided, by making their patients’ clinical data available for research.

## Discussion

### Principal Findings

Information about the requirements under which patients would make their treatment data available for research is important for any form of policy that regulates the secondary use of such data. This paper provides the results of a representative sample of German patients with cancer on general willingness and decisive requirements for sharing their data for research purposes and on the acceptance of consent models and expectations toward relevant stakeholders. The following are the main findings. First, we found an unprecedentedly high general willingness (810/838, 96.7%) to make clinical data available even after being informed about the potential risks of secondary use; however, relevant requirements included the following: ensuring a high level of data security, comparable data protection standards for data use abroad, and renewed consent at regular time intervals. Second, in contrast to previous studies, three-fourths of respondents (620/838, 73.9%) supported data use also by researchers in commercial companies. Third, the highest acceptance rate was found for a broad consent model (494/838, 58.9%), followed by data use by default (419/838, 50%); and specific consent for every study (327/838, 39%). Fourth, high expectations for physicians and fellow patients to support data sharing for research purposes were found.

To the best of our knowledge, this is the first representative study on attitudes toward the secondary use of clinical data and acceptance of consent models in combination with characteristics of data control and research utility.

### High General Willingness to Provide Clinical Data

#### Overview

An important finding of our study was the high willingness of patients with cancer to make their clinical data available for research purposes (810/838, 96.7%), either without any restrictions (527/838, 62.9%) or under certain conditions (283/838, 33.8%). Only 0.7% (6/838) of the participants generally refused to provide clinical data. A population-representative study in the United States found low proportions of general willingness (76%) [21], similar to representative studies in Germany in the contexts of the COVID-19 pandemic (65%) [22] and medical data including genetic data (56%) [13]. These different results suggest that patients with cancer are more willing to provide clinical data for medical research because they may either have benefited or hope to benefit from research. As potential beneficiaries of past studies, they may also feel greater responsibility than citizens and other patient groups to support research to help future generations of patients [11,23-25]. Although patients with cancer are not representative of all patients, we assume that they can hint well at the attitude of other patient groups with severe or rare diseases, such as leukodystrophies [26]. A study conducted in the United States shows slightly lower willingness among patients with cancer and survivors of cancer (71%) [27] than among the general population (76%) [21], which may point toward country-specific factors in the context of health systems and trust in institutions.

#### General Requirements: Data Security, Maximizing Data Use, and Transparency

The most relevant general requirements for supporting the secondary use of clinical data for research were high data security (486/838, 57.9%), maximizing data use (251/838, 29.9%), and information about research results that made use of patients’ clinical data (210/838, 25.1%). These findings indicate the relevance of the ability of data governance to protect clinical data, maximize accessibility (and usability) of data for research, and report transparently on the results of data use. These findings are largely consistent with previous literature that describes secure data use, public benefits through effective use by researchers, and transparency as important requirements for data sharing [3,8,14]. It may well be that participants value the reporting of results as an act of recognition and reciprocity. Suggestions for future set up of governance for secondary use of data to respond to the abovementioned requirements include appropriate safeguards to protect patient data; high degree of transparency regarding data use and benefits to society; and technical, organizational, and legal data infrastructure that enables researchers to maximize research benefits. Involving patients to better understand their concrete needs in designing these requirements for secondary use seems advisable [28].

#### Data Transfer Only to Countries With Comparable Data Protection Standard

Most participants stated that they would restrict their data to research in countries with data protection standards comparable with those in Germany (737/838, 87.9%), and a small minority of the participants was willing to provide data to other countries (75/838, 8.9%). This resonates with another German study with outpatients who generally support data donation in favor of public research institutions in EU countries with similar data protection standards (92%); only a minority of the participants approved data access to countries outside the EU (24%), which is a large share compared with our findings [6]. The high relevance of this aspect is consistent with studies of Canadian citizens [8,9]. However, further studies are needed to explore the exact kinds of misuse that make people fearful about international data transfers. Our study suggests that comparable data protection standards are a decisive requirement for patients. A suggestion to address this need is that policy makers and data initiatives explain well to patients what the additional benefit of multinational research is, what the specific risks are (eg, foreign government access and less ability to enforce rights), and how risks to data protection in these countries are mitigated. They are well advised to give patients the choice of whether to consent to data transfer to countries with low data protection standards.

#### Most Participants Support Data Use by Corporate Researchers

Low willingness of citizens to share data with the private industry has been reported in several studies [3,4]. This finding poses challenges to the biomedical research landscape, as many studies are conducted by companies or in cooperation with companies. In contrast, our results show that approximately three-fourths of the participants (620/838, 73.9%) were willing to make their clinical data available to company researchers. This is a much higher acceptance than in studies with German citizens [5] and outpatients [6], which reported that only a minority of those participants who agreed to data donation were willing to provide data to the industry (17% and 29%, respectively). A cross-country study found particularly low support for medical and genetic data sharing with for-profit researchers among German participants (22% compared with 32% on average across all countries) [29]. We hypothesize that willingness to share data with company researchers may change owing to experiences with a severe illness: patients with cancer may develop strong awareness of contributions by corporate researchers, possibly based on their experiences during their therapy. In addition, our questionnaire item included a brief explanation of the relevant contribution of industry to medical research and of industry as an important collaborator with public research institutions. We suppose the explanation increased the participants’ understanding and willingness to provide clinical data to the industry, which is consistent with a study examining public attitudes toward commercial data access, in which provision of information and deliberative methods increased willingness to share data [15]. In addition, our findings indicate that low willingness to share data with corporate researchers can be addressed through collaboration with public research institutions in public-private partnerships.

#### Renewed Consent Within Certain Time Intervals

The participants’ stance was divided on the duration of data use after initial consent is provided. Most participants (408/838, 48.7%) preferred to renew consent for broad research use after a period of 3 or 10 years. Only approximately one-third of the participants (243/838, 28.9%) preferred 1-time consent with unlimited duration of consent validity. In contrast, in a representative study of German citizens, more than half of the participants favored unlimited validity of consent (56%), and a minority favored consent validity of 5 years (17%) [5]. Our reported relatively high proportion of participants preferring renewed consent may have resulted owing to the following reasons. First, patients with cancer experience changing health conditions, leading to a subjective sensitivity to release clinical data without time limit. Second, our questionnaire explicitly mentioned risks of data release, possibly reducing the approval of unlimited data use. Third, the abovementioned study among German citizens asked for unlimited use for “data donation,” which can be understood as irrevocable by definition. To address this potential need for patients to renew consent, further studies should investigate the preferences using neutral wording.

### Broad Consent and Data Use by Default Was More Accepted Than Specific Consent—Research Benefits Partially Outweigh Loss of Control

#### Overview

Participants were presented with general information about 3 consent models (specific consent for every study; broad consent; and data use by default, with the option to opt out at any time). Specific consent is related to maximum informational control for patients, but less utility for research projects, whereas data use by default is associated with less informational control, but maximum utility for research projects. The broad consent model features moderate control and research utility (Table 2). The opportunity of being personally given information by health personnel is not available in the case of data use by default. Participants rated the level of acceptance for each consent model. The broad consent model received the highest acceptance rate (491/838, 58.6%), followed by data use by default (421/838, 50.2%) and the specific consent model with only a moderate acceptance rate (323/838, 38.5%). The relatively high acceptance rate for the broad consent model is consistent with the results of previous studies. Different study designs and minor deviations regarding the definition of consent procedures apply; therefore, comparisons should be considered cautiously. In total, 2 studies with a German patient sample and a large sample of Dutch patients found even higher acceptance rates in the context of health care–embedded biobanking and data donation (92%-93%) [5,10]. An earlier study of German patients (87%) [11] and a study of a smaller sample of US citizens (96%) [12] showed similar results. Our acceptance rates for each of the presented consent models were lower than those in other studies. This may be a consequence of the choice among 3 different models, rather than only 1, as presented in other studies. The low acceptance rates may also result from a trade-off decision between support for research and control over one’s clinical data. Previous studies have described this trade-off between control and research benefits as a relevant influencing factor in decision-making [12-16]. Accordingly, in our study, most participants (520/838, 62.1%) agreed to give up control if it increased the benefits of research. This finding is significant because most participants (804/838, 95.9%) believe in the benefits of secondary use for other patients. Evidence from other studies [11,23-25] and our findings not only suggest that research benefits partially outweigh the loss of control but also that they are a critical motivational aspect of making data available for research.

As none of the models achieved wide-ranging acceptance in our study, it is worth discussing whether a meta-consent model that allows participants to choose their preferred consent variants [30] accounts best for individual ways of balancing control and research benefits regarding consent models.

#### Preferred Framework Conditions for Providing Consent and Data Release

When asked for consent, participants expected brief and understandable written information (616/838, 73.5%) about data use and preferred their primary care physician as a venue for informed consent (528/838, 63%) over providing consent upon hospital admission (174/838, 20.8%). This finding is underpinned by a qualitative study in which support by health care professionals was seen as an important facilitator [8].

Our findings indicate that, regarding place and time (ie, where and when patients are informed and asked for consent), consent in the clinical context is preferred over consent before becoming a patient. This is consistent with the finding that patients prefer providing consent at hospitals (64%-76%) over providing consent outside the clinic [6]. However, another study concluded that the decision about making data available for research should be separated from the clinical context and anchored in everyday life [31]. Owing to possible age and disease effects, further studies should investigate the differences between the general population’s and patients’ acceptance.

When asked who should decide on data release when individual research projects apply for using participants’ clinical data after having personally released their treatment data for research purposes, approximately half of the participants (394/838, 47%) preferred a committee with experts and patient representatives over a committee with experts only or deciding for themselves. A suggestion to address this need is to involve patients in data access committees.

### Low Concerns and High Expectations

#### Low Level of Concern in General and About Discrimination

In our study, the proportion of participants who were concerned about the use of clinical data (101/838, 12.1%) was considerably lower than the findings of 2 surveys conducted in Australia among citizens (24%-25%) [32] and patients (24%) [33]. A study conducted in the United States found that privacy concerns had the strongest influence on individuals’ intentions to provide clinical data [34]. This discrepancy may be attributable to country-specific differences regarding trust in health care and government institutions [3] and the lack of experience with extensive data leaks or the misuse of clinical data in Germany. Participants’ concerns about discrimination owing to their cancer were very low (34/838, 4.1%).

#### False Expectations of Personal Benefit

Most participants (486/838, 57.9%) incorrectly expected personal benefits from making their clinical data available for research purposes—even though the wording of the questionnaire had been adjusted during the pretest phase. Another study found that more than one-fourth of German patients hoped for personal benefit (28%) after being asked for consent for secondary use of clinical data and biomaterial collected during routine care [11]. Owing to the severity of the disease, patients with cancer may be particularly prone to this false expectation of personal benefit from research with their health data, which is comparable with therapeutic misconception [35] in clinical trials. The study showed that the proportion of those holding false expectation decreased considerably after the modification of consent information material (12%). To reduce the risk of false expectations, particularly in vulnerable groups such as patients with severe illnesses, careful education about the unlikelihood of direct benefits from making their clinical data available for research purposes is needed.

#### High Expectations of Other Patients and Physicians

Our results indicate a clear expectation toward fellow patients (696/838, 83.1%) to support medical research with clinical data, which is consistent with a study conducted in Germany among outpatients (80%-90%) [6]. Interestingly, more participants expected their physicians to share clinical data for research (754/838, 89.9%) than to protect their clinical data under all circumstances (570/838, 68%). This is the first study to investigate the expectations toward physicians.

### Limitations

The recruited sample is largely representative of the population of patients with cancer in the federal state of Baden-Württemberg in age, gender, and cancer entity. However, we found that the educational level in our sample was higher than that of the corresponding age cohorts of the German population [36]. The educational level of the German population presumably applies to the group of patients with cancer and survivors of cancer. Owing to the topic of the survey, we suspect a self-selection bias correlating with high educational level. According to a study in the context of genetic research and biobanking, high educational level positively correlates with willingness to provide data [24]; consequently, our results may overestimate willingness to provide clinical data. In addition, false expectations of personal benefits from providing data for secondary use may have increased the participants’ willingness to share clinical data.

A considerable proportion of participants who had previously agreed to hypothetically make their clinical data available *without restrictions,* favored restricted use of their clinical data when asked about specific requirements such as data user, duration, and data use in other countries (Multimedia Appendix 9). We assume that the participants have not yet formed a strong opinion about sharing their clinical data. Hence, the general willingness to provide clinical data seems to measure an overall attitude toward secondary use, rather than the actual willingness to provide clinical data without restrictions for research purposes.

### Conclusions

Our study shows very high general willingness of patients with cancer to make their clinical data available for biomedical research purposes. However, the willingness to provide clinical data may be overstated owing to the above-average educational level of the respondents. For a considerable proportion of patients with cancer, willingness depends on certain requirements. In addition to the basic prerequisite of high level of data security and transparency in the use of the data, most patients shared the view that the data must not be used in countries with low data protection standards and that they should have the possibility to renew consent. In contrast to previous studies, the exclusion of use of data for private sector studies is not a requirement for most participants.

High willingness on the part of patients to accept loss of control over clinical data in favor of research benefits and request to maximize accessibility (and usability) of data for research were found. This is consistent with the acceptance of more research-friendly and low-control models, namely the broad consent model, followed by data use by default (with the option to opt out at any time). The striving for maximizing data use is also reflected by patients’ expectations toward physicians and other patients to support secondary use.

Policy makers are well advised to account for patients’ views when designing and implementing secondary use, with the aim to contribute to a socially legitimized culture of data sharing.

## Appendix

Multimedia Appendix 1 Questionnaire.

Multimedia Appendix 2 Participants’ requirements that are to be met to provide clinical data.

Multimedia Appendix 3 The acceptance of use of clinical data for biomedical research purposes in other countries.

Multimedia Appendix 4 Preferred duration of use of clinical data for biomedical research purposes.

Multimedia Appendix 5 Participants’ restrictions for the provision of their clinical data regarding different researcher groups.

Multimedia Appendix 6 Preferred context of providing informed consent.

Multimedia Appendix 7 Ways of obtaining information.

Multimedia Appendix 8 Decision on data release for individual research projects.

Multimedia Appendix 9 Conditional data release of participants initially stating to provide data without restriction.

## Abbreviations

EU: European Union

## Data Availability

The data sets generated and analyzed in this study are available on the web [37].
