# Supplementary material for: Patients’ Willingness to Provide Their Clinical Data for Research Purposes and Acceptance of Different Consent Models: Findings From a Representative Survey of Patients With Cancer
Source: J Med Internet Res. 2022 Aug 25;24(8):e37665. doi: 10.2196/37665 (PMC9459939; doi:10.2196/37665)
Supplement: Multimedia Appendix 1 [file jmir_v24i8e37665_app1.pdf]

⇒ **Wir bitten Sie den Fragebogen <u>vollständig</u> auszufüllen und die <u>Fragen in der angegebenen Reihenfolge</u> zu beantworten.**

Fragen zu Ihrem Vorwissen und Ihren Einstellungen

1. Üben Sie aktuell oder haben Sie in der Vergangenheit einen Beruf im Gesundheitswesen ausgeübt?

☐ Ja

☐ Nein

2. Haben Sie schon einmal als Probandin oder Proband an einer medizinischen Studie teilgenommen?

☐ Ja

☐ Nein

3. Bitte kreuzen Sie an, wie sehr Sie den folgenden Personen vertrauen.

	Kein Vertrauen	Geringes Vertrauen	Mittleres Vertrauen	Höheres Vertrauen	Volles Vertrauen	Weiß nicht
Ärztinnen und Ärzten, bei denen ich in Behandlung bin	☐	☐	☐	☐	☐	☐
Forscherinnen und Forschern an Universitäten/Universitätskliniken	☐	☐	☐	☐	☐	☐
Forscherinnen und Forschern in Unternehmen, die medizinische Forschung betreiben.	☐	☐	☐	☐	☐	☐

4. Bitte kreuzen Sie an, inwieweit Sie den folgenden Aussagen zustimmen oder nicht zustimmen.

	Stimme nicht zu	Stimme eher nicht zu	Teils-teils	Stimme eher zu	Stimme zu
Ich bin davon überzeugt, dass die meisten Menschen gute Absichten haben.	☐	☐	☐	☐	☐
Heutzutage kann man sich auf niemanden mehr verlassen.	☐	☐	☐	☐	☐
Im Allgemeinen kann man den Menschen vertrauen.	☐	☐	☐	☐	☐

5. Bitte kreuzen Sie an, inwieweit Sie den folgenden Aussagen zustimmen oder nicht zustimmen.

	Stimme nicht zu	Stimme eher nicht zu	Teils-teils	Stimme eher zu	Stimme zu	Weiß nicht
Ich möchte medizinische Forschung unterstützen.	☐	☐	☐	☐	☐	☐
Medizinische Forschung ist, verglichen mit anderen Forschungsbereichen, eine besonders wichtige Aufgabe für Deutschland.	☐	☐	☐	☐	☐	☐
Ich lege im Allgemeinen sehr großen Wert darauf, dass meine Daten nur unter höchsten Datenschutz-Standards verarbeitet werden.	☐	☐	☐	☐	☐	☐
Mir ist es im Allgemeinen sehr wichtig, die Kontrolle über meine Daten zu haben.	☐	☐	☐	☐	☐	☐
Wenn es der medizinischen Forschung nützt, ist mir die Kontrolle meiner Daten weniger wichtig.	☐	☐	☐	☐	☐	☐

⇒ **Bitte lesen Sie zunächst die Info-Box und beantworten Sie anschließend die folgenden Fragen.**

In Deutschland suchen täglich zahlreiche Patientinnen und Patienten **Kliniken oder Arztpraxen** auf. **Dabei fallen immer Daten an.** Diese Daten umfassen beispielsweise **Diagnosen, Informationen zum Krankheitsverlauf oder Laborwerte.**

Diese Daten werden im Folgenden **Behandlungsdaten** genannt, um sie von anderen Daten (wie beispielsweise Daten für Online-Banking oder aus sozialen Medien) zu unterscheiden.

---

6. Bitte kreuzen Sie an, inwieweit Sie den folgenden Aussagen zustimmen oder nicht zustimmen.

	Stimme nicht zu	Stimme eher nicht zu	Teils-teils	Stimme eher zu	Stimme zu	Weiß nicht
Mir ist es sehr wichtig, die Kontrolle über meine **Behandlungsdaten** zu haben.	☐	☐	☐	☐	☐	☐
Meine **Behandlungsdaten** sind schützenswerter als meine anderen Daten.	☐	☐	☐	☐	☐	☐

---

7. Wie würden Sie, ohne lange darüber nachzudenken, das Risiko durch die Verarbeitung der folgenden Informationen einschätzen?

	Kein Risiko	Niedriges Risiko	Mittleres Risiko	Hohes Risiko	Sehr hohes Risiko
Bankdaten	☐	☐	☐	☐	☐
Familienfotos im Internet	☐	☐	☐	☐	☐
Steuererklärung	☐	☐	☐	☐	☐
Behandlungsdaten (wie Diagnosen und Laborwerte) ohne persönliche Daten (wie Name, Adresse, Geburtsdatum)	☐	☐	☐	☐	☐

Ihre Erwartungen gegenüber Forschung mit Behandlungsdaten

⇒ **Bitte lesen Sie zunächst die Info-Box und beantworten Sie anschließend die folgenden Fragen.**

Behandlungsdaten können für Forschungszwecke verwendet werden.
Diese Behandlungsdaten werden **wahrscheinlich nicht nur der Forschung, sondern auch Patientinnen und Patienten in der Zukunft nutzen:** So könnten beispielsweise mithilfe der Forschungsergebnisse die Sicherheit von Medikamenten und die Versorgungsqualität in Krankenhäusern verbessert werden.

---

8. Bitte kreuzen Sie an, inwieweit Sie den folgenden Aussagen zustimmen oder nicht zustimmen.

	Stimme nicht zu	Stimme eher nicht zu	Teils-teils	Stimme eher zu	Stimme zu	Weiß nicht
Von der Forschung an meinen Behandlungsdaten erwarte ich eine bessere Gesundheitsversorgung **für mich persönlich.**	☐	☐	☐	☐	☐	☐
Von der Forschung an meinen Behandlungsdaten erwarte ich eine bessere Gesundheitsversorgung **für andere Patientinnen und Patienten.**	☐	☐	☐	☐	☐	☐
Wenn ich die Vorteile medizinischer Forschung nutze, dann **sollte ich persönlich** meine Behandlungsdaten **freiwillig** für Forschungszwecke zur Verfügung stellen.	☐	☐	☐	☐	☐	☐
Wenn alle die Vorteile medizinischer Forschung nutzen, dann **sollten alle Patientinnen und Patienten** ihre Behandlungsdaten **freiwillig** für Forschungszwecke zur Verfügung stellen.	☐	☐	☐	☐	☐	☐

⇒ **Bitte lesen Sie zunächst die Info-Box und beantworten Sie anschließend die folgenden Fragen.**

Wie in allen Bereichen, in denen Daten verarbeitet werden, kann auch bei Behandlungsdaten **nicht** ausgeschlossen werden, dass es zu **Datenschutzpannen kommt oder die Daten missbräuchlich verwendet werden.**
Auch ohne persönliche Daten (wie Name, Adresse, Geburtsdatum) ist es **theoretisch möglich unter großem Aufwand einzelne Personen** anhand der Behandlungsdaten **zu identifizieren**.

Die **Wahrscheinlichkeit** solcher Vorfälle gilt aktuell als **gering.**

---

9. Bitte kreuzen Sie an, inwiefern Sie der folgenden Aussage zustimmen oder nicht zustimmen.

	Stimme nicht zu	Stimme eher nicht zu	Teils-teils	Stimme eher zu	Stimme zu	Weiß nicht
Ich hätte große Sorge um die Sicherheit meiner Behandlungsdaten, wenn diese für medizinische Forschung verwendet würden.	☐	☐	☐	☐	☐	☐
Ich schätze das Risiko einer Identifizierung meiner Person durch Behandlungsdaten **nicht** höher ein als bei anderen Daten.	☐	☐	☐	☐	☐	☐

Freigabe der eigenen Behandlungsdaten für Forschungszwecke

⇒ **Bitte lesen Sie zunächst die Info-Box und beantworten Sie anschließend die folgenden Fragen. Bitte beachten Sie, dass die Beantwortung dieser Fragen keine tatsächliche Freigabe von Behandlungsdaten nach sich zieht.**

Stellen Sie sich nun vor, dass man Sie bei Ihrem nächsten Besuch in einer Arztpraxis oder einer Klinik fragt, ob Sie Ihre Behandlungsdaten für medizinische Forschungsprojekte bereitstellen wollen.

- Bei jedem Besuch in einer Arztpraxis oder Klinik werden mit Ihrem Einverständnis in den **kommenden Jahren** dann Ihre Behandlungsdaten über **jede Art von Erkrankung** gesammelt.

- **Ihre Behandlungsdaten werden nach Ihrer Erlaubnis in Datenzentren gespeichert.** Ihre persönlichen Informationen (wie beispielsweise Ihren Namen oder Ihre Adresse) werden nicht gespeichert. Ihre Behandlungsdaten werden mit einer **Identifikationsnummer** versehen, damit neue mit den bestehenden Daten zusammengeführt werden können.
Forscherinnen und Forscher erhalten Ihre Daten also **ohne persönliche Informationen**.

- Ihre Daten sollen für medizinische Forschungsprojekte verwendet werden, die **in der Zukunft** durchgeführt werden. Das bedeutet auch, dass der genaue **Forschungszweck noch unbekannt** ist.

---

10. Bitte wählen Sie eine Antwort aus und gehen Sie anschließend weiter zur angegebenen Frage.

☐ Ich würde meine Behandlungsdaten zu Forschungszwecken **nur unter bestimmten Bedingungen** zur Verfügung stellen.

⇒ **Bitte gehen Sie nun zu Frage 11 auf der nächsten Seite**

☐ Ich würde meine Behandlungsdaten zu Forschungszwecken **uneingeschränkt** zur Verfügung stellen.

⇒ **Bitte gehen Sie nun zu Frage 11 auf der nächsten Seite**

☐ Ich würde meine Behandlungsdaten zu Forschungszwecken **grundsätzlich nicht** zur Verfügung stellen.

⇒ **Bitte überspringen Sie die nächsten Fragen und gehen Sie zu <u>Frage 15</u>**

---

11. Wie lange sollten Ihre Behandlungsdaten verwendet werden dürfen bevor man Sie erneut um Erlaubnis fragen muss?

Bitte wählen Sie <u>eine</u> Antwort aus.

☐ Bis zu 3 Jahren

☐ Bis zu 10 Jahren

☐ Bis zu 30 Jahren

☐ Ohne Zeitbegrenzung

☐ Ich möchte bei *jeder einzelnen Verwendung* meiner Behandlungsdaten für medizinische Forschungszwecke angefragt werden.

---

12. Sowohl Forscherinnen und Forscher in öffentlichen Forschungseinrichtungen und in privaten Unternehmen leisten einen wichtigen Beitrag zur medizinischen Forschung. Manchmal lassen sich durch die Zusammenarbeit bessere Ergebnisse erzielen.

Bitte kreuzen Sie <u>alle</u> Personen an, denen Sie Ihre Behandlungsdaten für Forschungszwecke <u>nicht</u> zur Verfügung stellen möchten.

☐ Forschende Ärztinnen und Ärzte, bei denen ich in Behandlung bin

☐ Forscherinnen und Forschern an *Universitäten/Universitätskliniken*

☐ Forscherinnen und Forschern in *Unternehmen*, die medizinische Forschung betreiben

☐ Forscherinnen und Forschern in Forschungsprojekten, bei denen *Universitätskliniken mit Unternehmen* zusammenarbeiten

☐ *Alle Forscherinnen und Forscher dürfen meine Behandlungsdaten verwenden.*

13. Wenn Behandlungsdaten *in mehreren Ländern* gesammelt und genutzt werden, könnten voraussichtlich bessere Forschungsergebnisse erzielt werden. In welchen Ländern würden Sie Ihre Behandlungsdaten zur Verfügung stellen?

Bitte wählen Sie eine Antwort aus.

☐ In Deutschland

☐ In Deutschland und Ländern mit vergleichbaren Datenschutz-Standards

☐ Überall/weltweit, unabhängig von Datenschutz-Standards

---

14. Ich stelle meine Behandlungsdaten nur dann für Forschungszwecke zur Verfügung, wenn…

(Bitte kreuzen Sie alle Antworten an, die zutreffen.)

☐ ich für jede *einzelne Studie* angefragt werde, ob ich meine Behandlungsdaten freigebe.

☐ ich über die wichtigsten *Ergebnisse aller Studien informiert* werde, für die meine Behandlungsdaten verwendet werden.

☐ ich selber entscheiden kann, *welche Arten von Behandlungsdaten* (z.B. Informationen zu psycho-therapeutische Behandlung, Alkoholkonsum) geteilt werden.

☐ ich selber entscheiden kann, *welche Gruppen von Forscherinnen und Forschern* (z.B. aus Unternehmen) meine Behandlungsdaten verwenden.

☐ meine Behandlungsdaten möglichst vielen *Forschungsprojekten zur Verfügung* gestellt werden.

☐ alle Anstrengungen unternommen werden, um eine *möglichst hohe Datensicherheit* zu gewährleisten.

☐ keine *genetischen Daten* gesammelt werden.

☐ ich dafür *bezahlt* werde.

☐ *Sonstiges:* ________

☐ *Nichts davon.*
Ich stelle meine Behandlungsdaten ohne diese Einschränkungen zur Verfügung.

Ihre bevorzugte Art der Mitbestimmung

15. Möglichkeiten Ihre Behandlungsdaten für Forschungszwecke freizugeben

⇒ **Bitte lesen Sie zunächst die Info-Box.**

Es gibt verschiedene Arten, Patientinnen und Patienten zu **informieren und mitentscheiden** zu lassen, ob und wie sie ihre Behandlungsdaten für Forschungszwecke zukünftig zur Verfügung stellen möchten.

Auf der nächsten Seite werden drei Möglichkeiten vorgestellt.

Bitte kreuzen Sie für jede dieser drei Möglichkeiten an, inwieweit diese für Sie in Frage kommt oder nicht in Frage kommt.

<u>1. Möglichkeit:</u> Einwilligung für <u>einzelne</u> Studien

- ➔ Sie werden **<u>persönlich aufgeklärt</u>**.
- ➔ Sie haben eine **<u>hohe Kontrolle</u>** über Ihre Behandlungsdaten, da Sie **bei <u>jeder einzelnen</u> medizinischen Studie** entscheiden können, ob Sie Ihre Daten zur Verfügung stellen.
- ➔ **<u>Forschung wird erschwert / verhindert</u>** aufgrund eines erhöhten Verwaltungsaufwands.

Kommt für mich in Frage	Kommt für mich eher in Frage	Kommt für mich eher nicht in Frage	Kommt für mich nicht in Frage	*Weiß nicht*
O	O	O	O	O

<u>2. Möglichkeit:</u> Einmalige Einwilligung für <u>zukünftige</u> Studien

- ➔ Sie werden **<u>persönlich aufgeklärt.</u>**
- ➔ Sie haben eine **<u>geringe Kontrolle</u>** über Ihre Behandlungsdaten, da Sie **einmalig für <u>alle</u> zukünftigen medizinischen Studien** um Ihre Erlaubnis gefragt werden.
- ➔ **<u>Forschung wird erleichtert</u>** aufgrund eines niedrigeren Verwaltungsaufwands.

Kommt für mich in Frage	Kommt für mich eher in Frage	Kommt für mich eher nicht in Frage	Kommt für mich nicht in Frage	*Weiß nicht*
O	O	O	O	O

<u>3. Möglichkeit:</u> Verwendung für <u>zukünftige</u> Studien <u>ohne</u> Einwilligung

- ➔ Sie erhalten allgemeine Informationen, werden aber **<u>nicht persönlich aufgeklärt</u>**. Sie können dieser Nutzung **<u>widersprechen</u>**.
- ➔ Sie haben eine **<u>sehr geringe Kontrolle</u>** über Ihre Behandlungsdaten, da die Behandlungsdaten **<u>aller</u> Patientinnen und Patienten** für Forschungszwecke genutzt werden **<u>ohne um Erlaubnis</u> zu fragen**.
- ➔ **<u>Forschung wird deutlich erleichtert</u>** aufgrund eines besonders niedrigen Verwaltungsaufwands.

Kommt für mich in Frage	Kommt für mich eher in Frage	Kommt für mich eher nicht in Frage	Kommt für mich nicht in Frage	*Weiß nicht*
O	O	O	O	O

Ihre Erwartungen an Aufklärung und Freigabeprozess

16. Welche Informationsangebote finden Sie persönlich *am wichtigsten*, um mehr über die Verwendung von Behandlungsdaten zu erfahren?

Bitte wählen Sie <u>maximal drei</u> Informationsangebote aus.

- ☐ Umfassendes schriftliches Informationsmaterial
- ☐ Kurze schriftliche Zusammenfassung der wichtigsten Punkte in leicht verständlicher Sprache
- ☐ Website
- ☐ Erklärvideos
- ☐ Handy-App
- ☐ Persönliche Beratung durch Ärztinnen und Ärzte
- ☐ Persönliche Beratung durch speziell geschultes Personal
- ☐ Telefonische Beratung durch eine zentrale Hotline

- ☐ *Sonstiges:* ____
- ☐ *Nichts davon.*

17. Welche der folgenden Situationen finden Sie persönlich *am geeignetsten*, um über die Freigabe Ihrer Behandlungsdaten für Forschungszwecke zu entscheiden?

Bitte wählen Sie <u>eine</u> Situation aus.

☐ Bei meinem Hausarzt

☐ Bei der Aufnahme in einem Krankenhaus

☐ Bei einem Behördengang (z.B. bei Ausgabe des Personalausweises)

☐ Orts- und zeitunabhängig mit einer Handy-App oder einer Website

☐ *Sonstiges:* ____

☐ *Weiß nicht.*

18. Bitte kreuzen Sie an, inwieweit Sie den folgenden Aussagen zustimmen oder nicht zustimmen.

	Stimme nicht zu	Stimme eher nicht zu	Teils-teils	Stimme eher zu	Stimme zu	Weiß nicht
Ich erwarte von meinen behandelnden Ärztinnen und Ärzten, dass sie unter allen Umständen meine Behandlungsdaten schützen.	☐	☐	☐	☐	☐	☐
Ich erwarte von meinen behandelnden Ärztinnen und Ärzten wissenschaftliche Forschung zu unterstützen, indem sie, sofern ich dem zugestimmt habe, meine Behandlungsdaten aufbereiten und der Forschung zur Verfügung stellen.	☐	☐	☐	☐	☐	☐

19. Bitte kreuzen Sie alle Aussagen an, denen Sie zustimmen.

Sollten meine Behandlungsdaten für die Forschung gesammelt werden, dann …

☐ berichte ich möglicherweise meinen Ärztinnen und Ärzten weniger Details über meine Erkrankungen.

☐ vermeide ich möglicherweise Arztbesuche.

☐ *Nichts davon.*

---

20. Stellen Sie sich vor, dass Sie persönlich Ihre Behandlungsdaten grundsätzlich für Forschungszwecke freigegeben haben. Nun können Forscherinnen und Forscher die Nutzung Ihrer Behandlungsdaten für jedes ihrer <u>Forschungsprojekte einzeln beantragen.</u> <u>Wer</u> soll Ihrer Meinung nach entscheiden, für welche einzelnen Forschungsprojekte Ihre Behandlungsdaten verwendet werden dürfen?

Bitte wählen Sie <u>eine</u> Antwort aus.

☐ Ich selber als Patientin oder Patient

☐ Gremien mit Expertinnen und Experten

☐ Gremien mit Expertinnen und Experten, in denen die Meinung von Patientinnen und Patienten vertreten ist, beispielsweise durch Patientenvertreter.

☐ *Weiß nicht.*

21. Bitte kreuzen Sie an, inwiefern Sie den folgenden Aussagen zustimmen oder nicht zustimmen.

Wenn ich daran denke, dass meine Behandlungsdaten für Forschungszwecke verwendet werden, dann hätte ich große Sorge, dass …

	Stimme nicht zu	Stimme eher nicht zu	Teils-teils	Stimme eher zu	Stimme zu	Weiß nicht
ich aufgrund meiner Krebserkrankung diskriminiert werde.	☐	☐	☐	☐	☐	☐
Unternehmen meine Behandlungsdaten für etwas anderes als medizinische Forschung verwenden.	☐	☐	☐	☐	☐	☐
mein Arbeitgeber meine Behandlungsdaten missbräuchlich verwendet.	☐	☐	☐	☐	☐	☐
meine Krankenkasse meine Behandlungsdaten missbräuchlich verwenden.	☐	☐	☐	☐	☐	☐
Kriminelle meine Behandlungsdaten missbräuchlich verwenden.	☐	☐	☐	☐	☐	☐
meine Behandlungsdaten ins Ausland gelangen und dort missbräuchlich verwendet werden.	☐	☐	☐	☐	☐	☐
die Polizei meine Behandlungsdaten für Strafverfolgungszwecke verwendet.	☐	☐	☐	☐	☐	☐

Abschließende Fragen zu Ihrer Person

22. Bitte kreuzen Sie an:

	Sehr gut	Gut	Mittelmäßig	Schlecht	Sehr schlecht
Wie ist Ihr Gesundheitszustand im Allgemeinen?	☐	☐	☐	☐	☐

23. Welche Art von Krebserkrankung wurde bei Ihnen zuletzt festgestellt?

Bitte wählen Sie eine Antwort aus.

☐ Mundhöhle/Rachen
☐ Magen
☐ Darm
☐ Bauchspeicheldrüse
☐ Lunge
☐ Malignes Melanom der Haut/Schwarzer Hautkrebs
☐ Brustdrüse/Brustkrebs
☐ Gebärmutterkörper
☐ Prostata
☐ Niere
☐ Harnblase
☐ Non-Hodgkin-Lymphome (alle malignen Lymphome außer Morbus Hodgkin)
☐ Leukämien

☐ *Andere Krebserkrankung*

24. Nehmen Sie aktuell oder haben Sie jemals Angebote in Anspruch genommen, bei denen Sie sich mit Patientinnen und Patienten über diese Erkrankung austauschen konnten (beispielsweise Selbsthilfegruppen oder Online-Plattformen)?

☐ Ja

☐ Nein

---

25. Fühlen Sie sich anderen Patientinnen und Patienten mit dieser Erkrankung verbunden?

☐ Ja

☐ Nein

---

26. Wie ist Ihre Krankheitssituation?

Bitte wählen Sie <u>eine</u> Antwort aus.

☐ Krebsbehandlung erfolgreich abgeschlossen

☐ Nach Diagnosestellung (Therapie hat noch nicht begonnen)

☐ Aktuell in Erstbehandlung

☐ Erstbehandlung abgeschlossen

☐ Rückfall/Rückfallbehandlung

☐ Palliative Behandlung

☐ *Weiß nicht.*

27. In welchem Rahmen findet Ihre Behandlung bzw. Ihre Nachsorge aktuell hauptsächlich statt?

Bitte wählen Sie eine Antwort aus.

☐ Stationär im Krankenhaus

☐ Ambulant im Krankenhaus

☐ In einer onkologischen Arztpraxis

☐ In einer anderen (Fach)Arztpraxis

☐ *Aktuell findet keine Behandlung/Nachsorge statt*

28. Welches Geschlecht haben Sie?

☐ weiblich

☐ männlich

☐ *keine Angabe*

29. In welchem Jahr sind Sie geboren?

__ __ __ __ (bitte Geburtsjahr angeben)

30. Was ist Ihr höchster Bildungsabschluss?

- ☐ Kein Schulabschluss
- ☐ Grund-/Volksschulabschluss
- ☐ Hauptschulabschluss
- ☐ Realschulabschluss
- ☐ (Fach-)Hochschulreife/Abitur
- ☐ (Fach-) Hochschulabschluss

31. Beschäftigungsstatus

- ☐ in Beschäftigungsverhältnis / selbständig tätig
- ☐ gesundheitsbedingt nicht erwerbstätig
- ☐ im Ruhestand
- ☐ aus anderen Gründen nicht erwerbstätig

32. Haben Sie Kinder?

- ☐ Ja
- ☐ Nein

33. Wie hoch ist das gesamte verfügbare monatliche Netto-Einkommen Ihres Haushalts?

☐ Bis 2.000 Euro

☐ 2.001 – 3.500 Euro

☐ 3.501– 5.000 Euro

☐ Über 5.000 Euro

☐ *Keine Angabe*

Herzlichen Dank für Ihre Teilnahme!

⇒ **Bitte nutzen sie das <u>adressierte und vorfrankierte Kuvert</u> für die Rücksendung des Fragebogens.**

Gibt es noch etwas, das Sie uns mitteilen möchten?

Möchten Sie mehr über diese Studie erfahren? Weitere Informationen finden Sie unter diesem Link:
https://www.nct-heidelberg.de/forschung/nct-core-services/nct-epoc/research/lincdat.html

Oder scannen Sie diesen QR-Code auf der linken Seite.

Bei Fragen wenden Sie sich bitte an Anja Köngeter:
Tel.: 06221 56 38407; anja.koengeter@med.uni-heidelberg.de
