# Supplementary material for: Patients’ Willingness to Provide Their Clinical Data for Research Purposes and Acceptance of Different Consent Models: Findings From a Representative Survey of Patients With Cancer
Source: J Med Internet Res. 2022 Aug 25;24(8):e37665. doi: 10.2196/37665 (PMC9459939; doi:10.2196/37665)
Supplement: Multimedia Appendix 2 [file jmir_v24i8e37665_app2.docx]

**Multimedia Appendix 2: Participants’ requirements that are to be met in order to provide clinical data (n=838); multiple answer item**

|  | **Values, n(%)** |
| --- | --- |
|  |  |
| Every effort is made to ensure the highest possible level of data security. | 482 (57.51) |
| My treatment data is available for as many research projects as possible. | 254 (30.31) |
| I am informed about the main results of all studies for which my treatment data are used. | 208 (24.82) |
| No genetic data is collected. | 152 (18.13) |
| I can decide for myself what types of treatment data (e.g., information about psycho-therapeutic treatment, alcohol use) will be shared. | 146 (17.42) |
| I can decide for myself which groups of researchers (e.g. from companies) may or may not use my treatment data. | 141 (16.82) |
| I am asked for each individual study whether I want to release my treatment data. | 127 (15.15) |
| I get paid for it. | 11 (1.31) |
|  |  |
| None of the above. I provide my treatment data without these restrictions. | 190 (22.67) |
| Other | 9 (1.07) |
| Do not know/not answered | 10 (1.19) |
