# Supplementary material for: Patients’ Willingness to Provide Their Clinical Data for Research Purposes and Acceptance of Different Consent Models: Findings From a Representative Survey of Patients With Cancer
Source: J Med Internet Res. 2022 Aug 25;24(8):e37665. doi: 10.2196/37665 (PMC9459939; doi:10.2196/37665)
Supplement: Multimedia Appendix 3 [file jmir_v24i8e37665_app3.docx]

**Multimedia Appendix 3: The acceptance of use of clinical data for (bio-)medical research purposes in other countries (n=832)**

|  | **Values, n(%)** |
| --- | --- |
|  |  |
| In Germany | 149 (17.91) |
| In Germany and countries with comparable data protection standards | 591 (70.52) |
| Everywhere/worldwide, independent of data protection standards | 73 (8.77) |
| Not answered | 19 (2.28) |
