# Supplementary material for: Patients’ Willingness to Provide Their Clinical Data for Research Purposes and Acceptance of Different Consent Models: Findings From a Representative Survey of Patients With Cancer
Source: J Med Internet Res. 2022 Aug 25;24(8):e37665. doi: 10.2196/37665 (PMC9459939; doi:10.2196/37665)
Supplement: Multimedia Appendix 4 [file jmir_v24i8e37665_app4.docx]

**Multimedia Appendix 4: Preferred duration of usage of clinical data for (bio-)medical research purposes (n=832)**

|  | **Values, n(%)** |
| --- | --- |
|  |  |
| Up to 3 years | 181 (21.75) |
| Up to 10 years | 227 (27.28) |
| Up to 30 years | 10 (1.20) |
| Without time limit | 320 (38.46) |
| I would like to be asked about each and every use of my treatment data for medical research purposes. | 85 (10.22) |
| Not answered | 9 (1.08) |
