# Supplementary material for: Patients’ Willingness to Provide Their Clinical Data for Research Purposes and Acceptance of Different Consent Models: Findings From a Representative Survey of Patients With Cancer
Source: J Med Internet Res. 2022 Aug 25;24(8):e37665. doi: 10.2196/37665 (PMC9459939; doi:10.2196/37665)
Supplement: Multimedia Appendix 5 [file jmir_v24i8e37665_app5.docx]

**Multimedia Appendix 3: Participants’ restrictions for provision of their clinical data regarding different researcher groups (n=832)**

|  | **Values, n(%)** |
| --- | --- |
|  |  |
| All researchers are allowed to use clinical data | 532 (63.39) |
| Not allowed to use clinical data^b^ |  |
| My physicians | 56 (6.73) |
| Researchers at universities/university hospitals | 48 (5.77) |
| Researchers in companies that conduct medical research | 189 (22.72) |
| Researchers in research projects in which university hospitals collaborate with companies | 111 (13.34) |
| Not answered | 25 (3.00%) |
| ^b^ Multiple answers possible | |
