# Supplementary material for: Patients’ Willingness to Provide Their Clinical Data for Research Purposes and Acceptance of Different Consent Models: Findings From a Representative Survey of Patients With Cancer
Source: J Med Internet Res. 2022 Aug 25;24(8):e37665. doi: 10.2196/37665 (PMC9459939; doi:10.2196/37665)
Supplement: Multimedia Appendix 6 [file jmir_v24i8e37665_app6.docx]

**Multimedia Appendix 6: Preferred context of giving informed consent (n=838)**

|  | **Values, n(%)** |
| --- | --- |
|  |  |
| At my family doctor’s practice | 528 (63.00) |
| Upon admission to a hospital | 174 (20.76) |
| Location and time independent with a mobile app or website | 68 (8.11) |
| When going to the authorities (e.g. when issuing an identity card) | 3 (0.35) |
|  |  |
| Other | 17 (2.02) |
| Do not know/not answered | 48 (5.72) |
