# Supplementary material for: Patients’ Willingness to Provide Their Clinical Data for Research Purposes and Acceptance of Different Consent Models: Findings From a Representative Survey of Patients With Cancer
Source: J Med Internet Res. 2022 Aug 25;24(8):e37665. doi: 10.2196/37665 (PMC9459939; doi:10.2196/37665)
Supplement: Multimedia Appendix 7 [file jmir_v24i8e37665_app7.docx]

**Multimedia Appendix 7: Ways of obtaining information (n=838); multiple answer item (maximum 3)**

|  | **Values, n(%)** |
| --- | --- |
|  |  |
| Short written summary of the most important points in easy to understand language | 616 (73.50) |
| Personal consultation with doctors | 347 (41.40) |
| Website | 185 (22.07) |
| Personal advice from specially trained staff | 157 (18.73) |
| Comprehensive written information material | 124 (14.79) |
| Explanatory videos | 104 (12.41) |
| Mobile app | 42 (5.01) |
| Telephone advice through a central hotline | 30 (3.57) |
|  |  |
| None of the above | 37 (4.41) |
| Not answered | 5 (0.60) |
