# Supplementary material for: Patients’ Willingness to Provide Their Clinical Data for Research Purposes and Acceptance of Different Consent Models: Findings From a Representative Survey of Patients With Cancer
Source: J Med Internet Res. 2022 Aug 25;24(8):e37665. doi: 10.2196/37665 (PMC9459939; doi:10.2196/37665)
Supplement: Multimedia Appendix 8 [file jmir_v24i8e37665_app8.docx]

**Multimedia Appendix 8: Decision on data release for individual research projects (n=838)**

|  | **Values, n(%)** |
| --- | --- |
|  |  |
| Committees with experts in which the opinion of patients is represented, for example by patient representatives. | 393 (46.89) |
| Myself as a patient | 200 (23.86) |
| Committees with experts and without patient representatives | 185 (22.07) |
|  |  |
| Do not know/not answered | 60 (7.15) |
