# Supplementary material for: Patients’ Willingness to Provide Their Clinical Data for Research Purposes and Acceptance of Different Consent Models: Findings From a Representative Survey of Patients With Cancer
Source: J Med Internet Res. 2022 Aug 25;24(8):e37665. doi: 10.2196/37665 (PMC9459939; doi:10.2196/37665)
Supplement: Multimedia Appendix 9 [file jmir_v24i8e37665_app9.docx]

**Multimedia Appendix 8: Conditional data release of participants initially stating to provide data without restriction**

|  | Duration (n=823) | Countries (n=813) | Research Group (n=806) |
| --- | --- | --- | --- |
|  | Usage without time limit; values, n(%) | Worldwide usage; values, n(%) | All researchers allowed to use data; values, n(%) |
|  |  |  |  |
| Willingness to provide clinical data without restriction | 269 (51.43) | 67 (12.96) | 385 (75.79) |
| Willingness to provide clinical data under certain conditions | 48 (17.08) | 5 (1.81) | 137 (4.11) |
